# Position Error Compensation *via* a Variable Reluctance Sensor Applied to a Hybrid Vehicle Electric Machine

**DOI:** 10.3390/s100301918

**Published:** 2010-03-09

**Authors:** İhsan Ömür Bucak

**Affiliations:** Computer Engineering Department, Engineering Faculty, Fatih University, 34500, Istanbul, Turkey; E-Mail: ibucak@fatih.edu.tr; Tel.: +90-212-866-3300-5530; Fax: +90-212-866-3412

**Keywords:** position error compensation, variable reluctance sensor, hybrid electric vehicles, induction machine, chi-square test

## Abstract

In the automotive industry, electromagnetic variable reluctance (VR) sensors have been extensively used to measure engine position and speed through a toothed wheel mounted on the crankshaft. In this work, an application that already uses the VR sensing unit for engine and/or transmission has been chosen to infer, this time, the indirect position of the electric machine in a parallel Hybrid Electric Vehicle (HEV) system. A VR sensor has been chosen to correct the position of the electric machine, mainly because it may still become critical in the operation of HEVs to avoid possible vehicle failures during the start-up and on-the-road, especially when the machine is used with an internal combustion engine. The proposed method uses Chi-square test and is adaptive in a sense that it derives the compensation factors during the shaft operation and updates them in a timely fashion.

## 1. Introduction

Motion control is a sub-field of automation, in which the position and/or velocity of machines are controlled using some type of device such as a hydraulic pump, linear actuator, or an electric motor, generally a servo. Motion control system provides precise control of the movement of various actuating elements of a device or system. Motion control is an important part of robotics and CNC machine tools, however it is more complex than in the use of specialized machines, where the kinematics are usually simpler. Motion control is widely used in the packaging, printing, textile, automotive and assembly industries. There are a number of extensive works that can be found in literature related to the motion control applications as some described in [1–8]. For example, in [1] Yano *et al*. considered control of a liquid container’s rotational motion along an inclined transfer path, paying special attention to the suppression of sloshing (liquid vibration) during acceleration and deceleration. This system is useful for saving space in factories and optimizing foundry processes. Su *et al*. in [2] designed to achieve high precision motion control against the variation of load and parameters. In another application in [3], Tian *et al*. presented a neural network approach for the motion control of constrained flexible manipulators. Leonard and Krishnaprasad in [4] discussed motion control of an autonomous underwater vehicle in the event of an actuator failure with an adaptive feature in the feedforward path. Bucak *et al*. in [5] aimed to control the motion of a horizontally moving vehicle mass on a rail, when it is subject to friction, under the influence of a controlled compressed spring. Hancock *et al*. in [6] used yaw motion control due to the inefficient and intrusive control systems when those systems utilize some form of brake or throttle intervention to generate a yaw moment and control wheel slip. Cao *et al*. in [7] proposed an active suspension control system for an half-vehicle model as part of automotive motion control systems to explore the nature of the effect of vehicle speed changes by introducing vehicle pitch angle as a system state. Finally, Raimondi and Melluso in [8] considered a fuzzy closed loop motion control for a cooperative system of automated passenger vehicles (*i.e.*, platoon) to ensure the stabilization of each vehicle in the planned trajectory and comfort of the human body during the motion, and guarantee low values of longitudinal and lateral accelerations. A motion controller generates the set points (the desired output) and closes the position feedback loop. A feedback sensor such as an optical encoder, resolver, or Hall-effect device is used to return the position of the actuator to the motion controller in order to close the position control loop [9,10]. Therefore, a rotational position is monitored using a rotor having vanes, teeth, or slots coupled to a revolving shaft, which transmits motion, for interacting with the feedback sensor which is positioned across from the toothed wheel and is used to sense the teeth as the wheel rotates. Modern feedback sensors almost exclusively use optical and magnetic sensors to convert the shaft’s angle to digital form. The magnetic sensors can be a Hall-effect element based sensors, or a passive electromagnetic device known as the Variable Reluctance (VR) sensor that generates AC voltage that increases with the shaft rpm (revolutions per minute) as the teeth pass through the sensor’s magnetic field [11]. No absolute position information is conveyed. A signal processing unit interprets the output of the sensor to infer position indirectly and provides a signal indicative of the absolute position of the shaft.

Magnetic sensors are largely applied to detect position in automotive systems. The conversion of speed or position to a magnetic signal enables non-contact magnetic detection that is unsusceptible to contamination and wear. For instance, magnetic detectors can be used to measure wheel and transmission speed, crank and cam shaft position for engine timing, throttle valve position for air intake, steering wheel position, pedal position, fluid level, chassis height, and in electronic door locks [12]. Recently, magnetic sensors have been increasingly replacing inductive sensors and resistive potentiometers used today in automotive position and speed detection as a choice of sensor because of their low cost.

A wheel has teeth disposed radially along the periphery of the wheel at a predetermined angular spacing and is mounted on the revolving shaft that transmits motion. The wheel has a first tooth and a second tooth positioned a meaningful distance apart from the first tooth. Ideally, the position of the second tooth, relative to the first tooth is indicated by an angular displacement. In this work, the wheel has a tooth every 20 degrees, with one tooth missing. Since one revolution is comprised of 360 degrees of shaft rotation, there will be 17 teeth excluding one missing for each shaft revolution. The missing tooth on the wheel indicates a fixed absolute position of the wheel. This missing tooth marker is used to synchronize the control of the machine dependent on this fixed position. Using the signal difference caused by the missing tooth, the sensor can provide the signals for the control unit to obtain shaft position in degrees and velocity in rpm. If the displacement causes an error, the feedback sensor will produce an erroneous result. This can never be tolerated for systems that rely on an accurate speed profile [13,14].

In order to determine the shaft velocity, only Δ*T* time values are actually measured. Each *Δθ* value is assumed to be known from the wheel design. Moreover, all *Δθ*’s are equally placed so that each computed velocity equals a constant (determined by a rotational arc covered by each measuring interval) divided by the measured time Δ*T* required to pass through the arc. The latter statement is best expressed by the following formula for *i*th selected rotation interval or the shaft revolution:

(1) $$V_{i}=\frac{{\Delta \theta }_{i}}{{\Delta T}_{i}}$$

where *V_i_* is the initial (uncorrected measured) while in the coast down mode. The corrected velocity V_i_^*^ is defined as:

(2) $$V_{i}^{*}=C*V_{i}$$

where *C* is chosen as the correction coefficient or factor. The measured values for both Δθ and Δ*T* must be measured sufficiently accurately to provide the sensitivity required to detect such small velocity changes. Deviations of the actual angles from the target values due to the non-uniform positioning of the wheel during manufacture process will result in erroneous velocities, and hence accelerations. If the angular error is sufficiently large, the erroneous values of velocity and acceleration can cause inaccurate system functioning or malfunction.

This paper is based on a method using the chi-square test that compensates for wheel profile irregularities in measured shaft positions resulting from errors in the physical placement of target teeth or position markers. These errors are known as position errors and a significant source of such irregularity in determining the rotational arcs during each measuring interval. During manufacture of a wheel, errors occur between the desired and actual positions for position markers on the wheel. Any deviation of the actual angle *Δθ* from the target value results in velocity and acceleration errors. The degree of impact that a given wheel error has on the acceleration calculation is strongly rpm dependent and the error can be fixed.

The proposed method is an adaptive one in a sense that it compensates for the wheel profile irregularities that derives the compensation factors during actual machine operation. The compensation factors are updated in a timely fashion.

The remaining of the paper is arranged as follows. Section 2 describes the proposed sensor, their operation and properties. Section 3 briefly explains the basics of the chi-square test, the calculation of the test statistic and interpretation of the test results. Section 4 describes the chi-square based iterative error compensation method, and studies an example of an induction machine by applying the method under the effect of viscous friction and presents the simulation results. Section 5 presents the experimental results of the actual system. Finally, conclusions are drawn in Section 6.

## 2. Variable Reluctance Sensor

**Figure 1.**
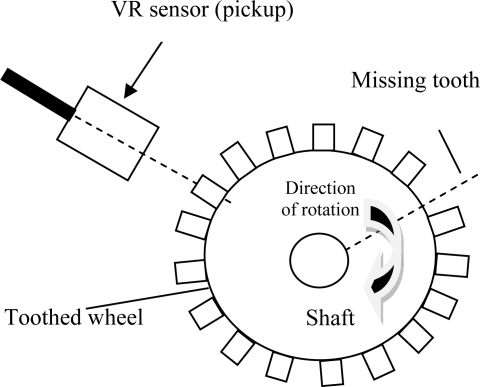
Variable Reluctance (VR) sensor that senses movement of the toothed wheel past point of sensor.

Figure 1 shows a VR sensor that senses movement of the toothed wheel past point of sensor. A VR sensor is used to measure position and speed of moving metal components. This sensor consists of a permanent magnet, a ferromagnetic pole piece, a pickup coil, and a rotating toothed wheel. VR sensors are often referred to a ‘passive’ magnetic sensor because it does not need to be powered. VR sensors are ‘self generating’. The variable reluctance wheel speed sensor is basically a permanent magnet with wire wrapped around it. It is usually a simple circuit of only two wires where in most cases polarity is not important. The physics behind the operation include magnetic induction.

Advantages of the VR sensor can be summarized as follows: low cost, robust proven speed and position sensing technology, self-generating electrical signal which requires no external power supply, fewer wiring connections which contribute to excellent reliability, and finally meeting a wide range of output, resistance, and inductance requirements so that the sensor can be tailored to meet specific customer requirements.

As the teeth pass through the sensor’s magnetic field, the amount of magnetic flux passing through the permanent magnet and consequently the coil varies. When the tooth gear is close to the sensor, the flux is at a maximum. When the tooth is further away, the flux drops off. The moving target results in a time-varying flux that induces a voltage in the coil, producing an electrical analog wave. The frequency and voltage of the analog signal is proportional to velocity of the rotating toothed wheel. Each passing discontinuity in the target causes the VR sensor to generate a pulse. The cyclical pulse train or a digital waveform created can be interpreted by a signal processing unit equipped with the required instrumentation.

One significant advantage that VR sensors offer is their low cost; coils of wire and magnets are relatively inexpensive. However, the low cost of the sensor is partially offset by the cost of the additional signal processing unit needed to recover a useful signal. Since they are currently used in wide range of automotive applications including engine and transmission, in this work, an application that already uses the VR sensing circuitry for engine and/or transmission has been chosen to infer, this time, the indirect position of the electric motor in the considered parallel HEV application. An alternative but more expensive technology is Hall-effect sensor.

VR sensors can be made to operate at temperatures in excess of 300 °C. Such high temperature applications include sensing the turbine speed of a jet engine, and engine cam shaft and crankshaft position control in an automobile. VR sensor is well-suited for a variety of other industrial applications, such as conveyer belts, truck, construction equipment, railroad and marine transmissions, automatic transmission in vehicles, All Terrain Vehicles (ATV) tachometer sensors, and ABS brake systems for wheel slip and traction control.

## 3. Chi-Square Test

### 3.1. Introduction

The chi-square test is one of the most commonly used methods for comparing frequencies, distributions, or proportions. The chi-square test is a statistical method used to determine if observed data deviate from those expected under a particular hypothesis. The chi-square test is also referred to as a test of a measure of fit or “goodness of fit” between data. Typically, the hypothesis tested is whether or not two samples are different enough in a particular characteristic to be considered members of different populations. The distribution of the test statistic under the null hypothesis fits the theoretical chi-square distribution. This means that once we know the chi-square test statistic, we can calculate the probability of getting that value of the chi-square statistic [15].

The chi-square analysis is used to test the null hypothesis (*H_0_*), which is the hypothesis that states there is no significant difference between expected and observed data. *H_0_* is either accepted or rejected after the value of chi-square is compared to a probability distribution. The significance of the chi-square statistic is often given in terms of a *p* value that is an indication of the likelihood of obtaining a result. chi-square values with low probability lead to the rejection of *H_0_* and it is assumed that a factor other than chance creates a large deviation between expected and observed results. As with all non-parametric tests (that do not require normal distribution curves), chi-square tests only evaluate a single variable, thus they do not take into account the interaction among more than one variable upon the outcome [15].

### 3.2. Calculation of the Test Statistic

The test statistic is calculated by taking an observed number (*O*), subtracting the expected number (*E*), then squaring this difference:

(3) $$X^{2}=\Sigma \frac{{\left[O-E\right]}^{2}}{E}$$

The larger the difference between observed and expected, the larger the deviation from the null hypothesis, or the larger the test statistic, becomes. Squaring the differences makes them all positive. Each difference is divided by the expected number, and these standardized differences are summed. The test statistic is conventionally called a “chi-square” statistic [15].

The general form for a test of a hypothesis concerning multinomial probabilities is given as *H_0_* : *p_1_* = *p_1,0_*, *p_2_* = *p_2,0_*,…, *p_k_* = *p_k,0_*, where *p_1,0_*, *p_2,0_*,…, *p_k,0_* represent the hypothesized values of the multinomial probabilities. The null hypothesis *H_0_* is established to indicate that the data follow the specified distribution. In this case, the test statistic is calculated by [16]:

(4) $$X^{2}=\Sigma \frac{{\left[n_{i}-E\left(n_{i}\right)\right]}^{2}}{E\left(n_{i}\right)},$$

where *E*(*n_i_*) = *np_i,0_* indicates the expected number of outcomes of type/class *i* assuming that *H_0_* is true, and *n* is a total sample size. It should be noted that the test statistic above has been defined under the following assumptions [16]:

1. A multinomial experiment is conducted. This is generally satisfied by taking a random sample from the population of interest.
2. The sample size *n* will be large enough so that for every cell, the expected cell count *E*(*n_i_*) will be equal to 5 or more.

The sample size of our measuring/test examples is 18, which is plentiful. Therefore the observed accuracy of the considered hypothesis over the measuring examples will be assumed at least not a poor estimator of its accuracy over future examples. Furthermore, variance in the estimate will not be greater because of the size of the set of test examples [17].

The shape of the chi-square distribution depends on the number of degrees of freedom, *i.e.*, the test statistic follows, approximately, a chi-square distribution with (*k-c*) degrees of freedom where *k* is the number of non-empty bins and *c* = 1 if the sample sizes are equal and *c* = 0 if they are not equal [18].

### 3.3. Interpretation of the Test Results

Assume a critical value and a number called degrees of freedom for the chi-square test. Critical values for the chi-square are determined from a statistical table based on the significance level at which the test is being performed. If the calculated chi-square value is equal to or greater than this critical value, it can be concluded that the probability of the null hypothesis being correct is some very small probability or less. If a calculated value is greater than the critical value, then the null hypothesis is rejected, and it is concluded that there is a significant difference between the observed and expected distributions.

## 4. Position Error Compensation Using Chi-Square Test

### 4.1. The Effect of Viscous Friction

As the velocity changes nonlinearly with the distance in coasting mode, exhibition of this nonlinear behavior has been effectuated by considering, at least, a simple model of the friction with a viscous term that is proportional to the velocity. Hence a rotational system that is an induction motor attached to a revolving shaft under the effect of viscous friction will be considered.

The system equation of motion is:

(5) $$J\frac{d\omega }{\mathrm{dt}}+b\omega =T_{s}(t)$$

where *T_s_*(*t*) is the torque the motor applies in N.m/rad; *J* is the shaft inertia in kg.m^2^/rad^2^; *b* is the viscous friction of coefficient in N.m.sec; *ω* is the angular position in radians and *dω/dt* is the angular velocity in rad/sec. This is a linear 1^st^ order ordinary differential equation (ODE) with constant coefficients. The complete response is the sum of the homogeneous term and the forced term:

(6) $$\omega (t)=\omega_{h}\;(t)+\omega_{f}\;(t)$$

The homogeneous term is due to the initial conditions in the system. In this case, a non-zero initial condition *ω* (*t* = 0) means that the wheel had been spinning before application of the torque by the motor at *t* = 0. The homogeneous term decays exponentially to zero (“spin-down”) if the system is stable. The system time constant *τ* can be obtained from the solution of the homogeneous term *ω_h_*(*t*) = *A*e*^−t/τ^* as follows:

(7) $$\tau =\frac{J}{b}$$

Note that the unknown coefficient *A* is to be obtained from the initial conditions in the homogeneous part of the response.

The forced term is due to the excitation or input of the system, as the name implies. The forced response can be found by firstly applying a step input to the system, and then by allowing the system to elapse a sufficiently long time (*i.e.*, as *t* → ∞) so that the angular velocity should be able to approach a constant value. This long-term constant value is called the “steady-state” response of the system and is found:

(8) $$\omega (\infty )=\omega_{f}\;(t)=\frac{T_{0}}{b}$$

where the unit for *ω*(∞) is 1/s or Hz.

The complete response is, therefore:

(9) $$\omega (t)=\omega_{f}\;(t)+\omega_{h}\;(t)=\frac{T_{0}}{b}+{\mathrm{Ae}}^{-t/\tau }$$

All that is left is to find the unknown coefficient *A*. Since the coefficient appears in the homogeneous part of the response, it must be determined by the initial conditions. It is assumed that the shaft was initially nonzero, *i.e.*, *ω_0_* = *ω* (*t* = 0) ≠ 0. The following is found through substitution:

(10) $$\omega_{0}=\frac{T_{0}}{b}+A\Rightarrow A=\omega_{0}-\frac{T_{0}}{b}$$

Replacing *A* in Eq. (9) with that of Eq. (10), and rearranging the resultant brings about the complete solution as follows:

(11) $$\omega (t)=\omega_{0}e^{-t/\tau }+\frac{T_{0}}{b}\left(1-e^{-t/\tau }\right)$$

It is important to note that if *ω*_0_ < *ω* (∞), the response “spins up” or accelerate to the steady-state from the initial condition, otherwise (*i.e.*, *ω*_0_ > *ω* (∞)) the response “spins down” or decelerate to the steady-state from the initial condition. The latter is the case that is definitely interesting to see how the motor-shaft system with viscous friction spins down from the initial condition when allowed to coast for the method to perform the measurements; *i.e.*, this event in whole can be summarized as deceleration/coast-down test due to the viscous-frictional drag.

### 4.2. The Method

The compensation factors are made for systematic irregularities arising from the position errors. Speed and load change will also change the compensations dynamically [19–21]. Therefore, an adaptive method of error compensation is desired. The method will be more accurate if the compensation factors are updated in a timely fashion. The method achieves error compensation by measuring time between the passages of position markers during rotation of the machine. Since most non-uniformity of the machine rotation is caused by the machine’s start-up that makes the wheel slip for an instant before it grabs the track, the method performs measurements with an unpowered, coasting wheel. Measuring encoded pulse trains directly and converting them into data streams during coasting enables to accurately quantify undesired speed fluctuations of rotating components, that is, eliminates the traditional speed term from the differential equation of motion of a freely coasting wheel, thereby eliminating a whole set of potential error sources, and improving the sensitivity and accuracy of time measurements [22–24]. Because the VR sensor is already used primarily to sense the engine cam shaft and crankshaft position control and operating the machine in an unpowered, coasting wheel mode is the most common approach for this action, it is also a practical approach to take advantage of the coasting to concurrently perform the measurements of the electric motor that is another rotating system with another VR sensor. For any parameter *x,* a general compensation factor equation is defined as follows:

(12) $$x_{\mathrm{corrected}}=x_{\mathrm{uncorrected}}\times \mathrm{CF}$$

where *CF* denotes the compensation factor. Therefore, the compensation factor based on a shaft angle for *i*th selected tooth is:

(13) $${\mathrm{CF}}_{i}=\frac{20{}^{\circ}}{{\Delta \theta }_{i_{\mathrm{measured}}}}$$

where 20° represents ideal geometry, or based on the measured time, the correction factor can be given as:

(14) $${\mathrm{CF}}_{i}=\frac{{\Delta T}_{f}}{{\Delta T}_{i_{\mathrm{measured}}}}$$

Assume that an actual velocity for each delta time interval is equal to an average velocity per revolution as follows:

(15) $$\frac{20{}^{\circ}}{{\Delta T}_{f}}=\frac{360{}^{\circ}}{\Sigma_{\mathrm{cyc}}({\Delta T}_{i_{\mathrm{measured}}})}$$

where Δ*T_f_* represents a fixed delta time when it is assumed that all the teeth around the wheel is equally placed with no manufacture error. Substituting Δ*T_f_* from (15) into (14) yields:

(16) $${\mathrm{CF}}_{i}=\frac{{\Delta T}_{f}}{{\Delta T}_{i_{\mathrm{measured}}}}=\frac{\Sigma_{\mathrm{cyc}}\left({\Delta T}_{i_{\mathrm{measured}}}\right)}{18*{\Delta T}_{i_{\mathrm{measured}}}}.$$

In the method, there are always two chi-square (*X^2^*) values to be computed. The first value is intended to set a critical value *X_α_* with a certain significance level. This value is critical to estimate how closely an observed distribution matches an expected distribution. Note that the farther the observed numbers are from their expected value, the larger *X^2^* will become. That is, large values of *X^2^* imply that the null hypothesis is false [16]. Therefore, the computed second value of *X^2^* should be less than the critical value for the null hypothesis not to be rejected. That also means that each time the probability of the null hypothesis being correct is guaranteed with an increasing probability. The purpose of comparing the second value with the first one is to obtain a single set of compensation factors for considered shaft revolution. The smaller *X^2^* value is assigned the first value during the computations of the next shaft revolution. These steps are repeated until a final set of compensation factors is obtained through the computations of *N* complete shaft revolutions. Once the computations of the entire shaft revolutions are completed, the final set of compensation factors obtained according to the algorithm will become a unique set of compensation factors that will represent the most accurate velocity, hence acceleration, and compensate for irregularities arising from position errors.

The chi-square test based compensation method does not require a continuous check of the manufacturer tolerances against the compensation factors during the computations of *N* complete shaft revolutions. The use of predetermined critical chi-square value does not need such consistency check since the null hypothesis may well be established based on the tolerances.

### 4.3. The Algorithm

**Figure 2.**
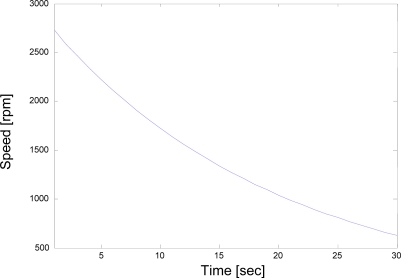
Simulated coast-down curve.

The following is the flow chart of the algorithm used for the error compensation (see next page). In the algorithm, the centre of the set comprising one complete shaft revolution is also known as the (current) measured time and will be used as the observed number in the chi-square equation in (3). Figure 2 shows the simulation of the rpm vs. time curve (coast-down test) which assumes that *T_0_* = 2 N·m, *J* = 4 kg.m^2^, *b* = 2.0 N·m·s, and *ω_0_* = 300 rad/s.

**Figure:**
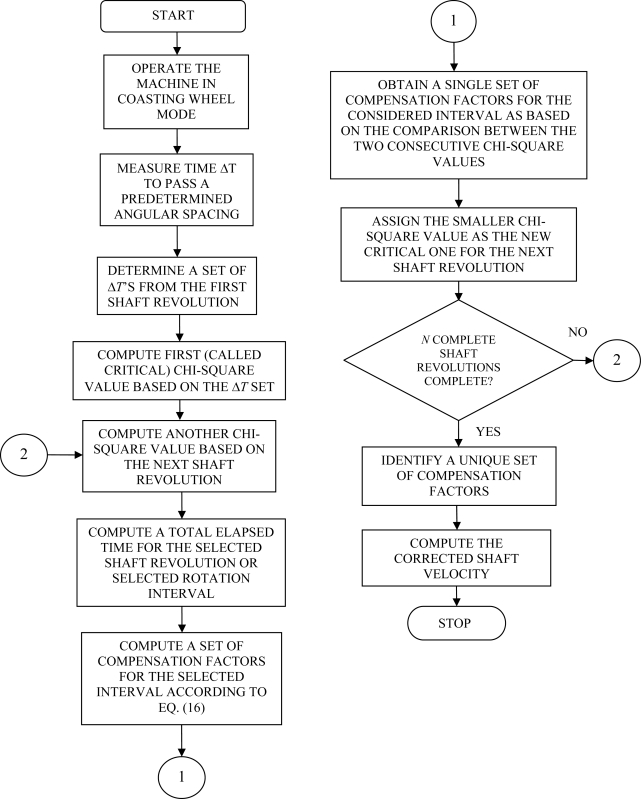


**Figure 3.**
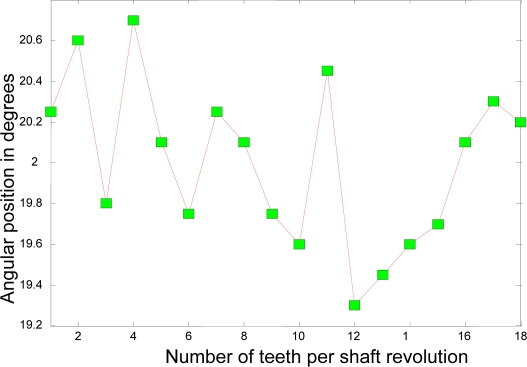
Angular delta shaft position vector before compensation.

**Figure 4.**
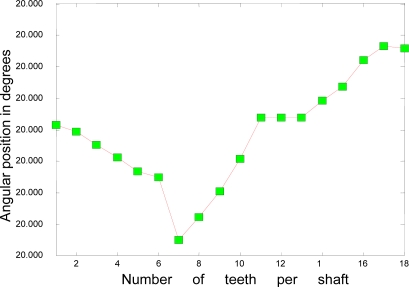
Angular delta shaft position vector corresponding with unique set of compensation factors.

**Figure 5.**
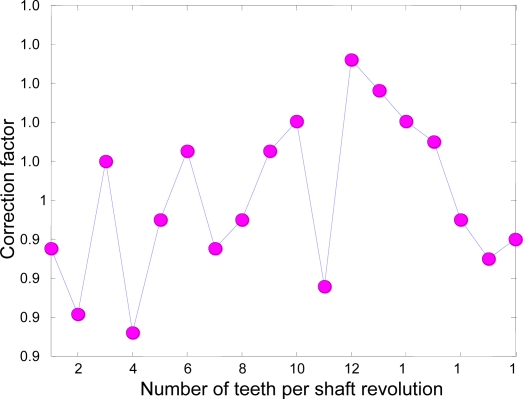
Unique set of compensation factors through the search of *N* complete shaft revolutions.

**Figure 6.**
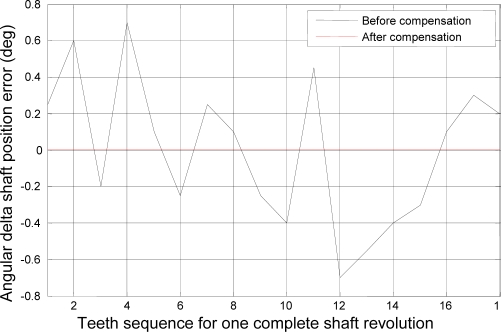
Angular delta shaft position error vector before- and after- compensation.

**Figure 7.**
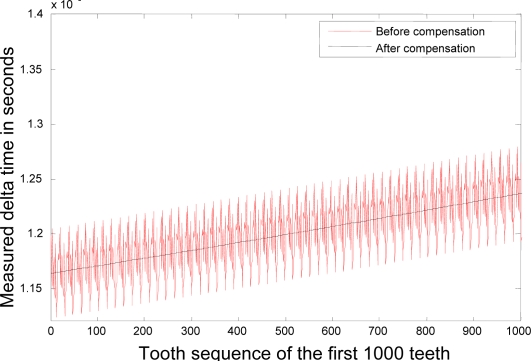
Measured time Δ*T* before- and after- compensation.

In this simulation the time interval was 0 ≤ *t* ≤*T* = 30 and the time step was Δ*t* = 0.001, so the figures represent 3834 *Tooth counts for N complete shaft revolutions* which is a product of 18 ‘*Number of teeth per shaft revolution*’ and 213 ‘*N complete shaft revolutions*’ where *N* is 213. Figures 3 and 4 depict the rotational angular delta position vectors before- and after- the compensation algorithm are applied, respectively. Figure 5 shows the unique set of compensation factors obtained through the algorithm for one complete shaft revolution. Figure 6 shows the angular delta shaft position error vector before- and after- the compensation algorithm are applied. Finally, Figure 7 shows the measured time for both before- and after- the algorithm are applied.

## 5. Experimental Results

**Figure 8.**
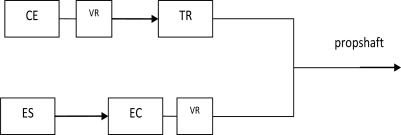
Structure of the Parallel system considered in the experiment. The letters indicate: CE: Internal combustion engine, EC: Energy conversion unit (Motor/Generator), ES: Energy storage unit (Battery), TR: Transmission, VR: Variable Reluctance sensor and its signal processing unit.

A simplified block diagram of the Parallel HEV system used in the experiment is shown in Figure 8.

**Figure 9.**
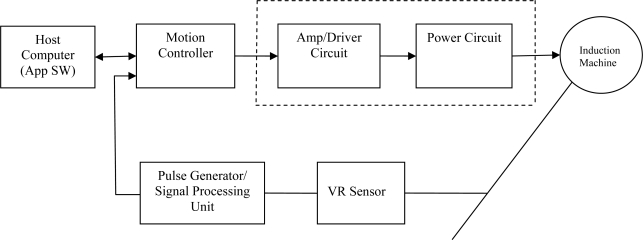
Main components of the proposed experimental system.

Figure 9 shows the picture of the proposed experimental system to illustrate the main components of its structure.

The controller directly outputs the signal for the power circuit and accepts a digital waveform of the position information (VR sensor signals) from a signal processing unit equipped with the required instrumentation. Signal processing unit produces a pulse train consisting of pulses synchronized with the passage of the leading edges of the toothed wheel past the sensor. VR sensor block includes the sensor pickup and the toothed wheel as shown in Figure 1. The host computer determines how the motion profile looks and how certain events trigger and influence it. It also contains instructions executed by the motion controller. With these instructions, the motion controller creates motion profiles. Based on these profiles, the controller sends signals through an amplifier, or motor drive, to the motor. As the motor turns, the feedback device – here the VR sensor as a position sensor- delivers position information through signal processing unit back to the controller to close the control loop. Therefore, the motion controller knows the position of the motor as the measured position of the machine and uses the measured signal and the reference signal to control the position or speed.

An AC induction motor has been chosen as an actual system for experimenting purposes. AC induction motors are the most common motors used in industrial motion control systems, as well as in main powered home appliances. Recently, they have also been increasingly one of the most demanding machines in HEV applications. Because the operating point of the traction engine does not stay in the most efficient operating range in vehicle applications. Series and parallel HEVs are known to have a 10 to 30 percent improvement in fuel economy by choosing the operating point within a higher efficiency range. Therefore, many automotive manufacturers have developed their own design using the induction machine as motor and/or generator, targeting the HEV system applications.

In an automotive industry, electromagnetic VR sensors have been extensively used to measure engine position and speed through a toothed wheel mounted on the crankshaft. However, in this work, the VR sensor has been applied to correct the position of the electric motor, mainly because it may still become critical in the operation of HEVs to avoid possible vehicle failures during the start-up and on-the-road, especially when it is used with an internal combustion engine. Excess acceleration, excess deceleration, and unintended vehicle acceleration as hazards need to be detected and mitigated within the required response time in the case of HEVs.

Single point of failure in velocity or accelerator may lead to overestimated driver request for acceleration, or underestimated wheel torque acceleration, or to detection and estimation which occurs too late and exceeds required response time, or too early or too sensitive. False detection of single point of velocity failure due to the engine speed, motor or generator speed may have the potential effect of false detection of the vehicle speed especially at low speeds developing in the form of creep torque for engine, and motor in generator mode. Potential effect of failing to detect single point of velocity failure may be to potentially disable detection of excess acceleration and excess deceleration, and detection of wheel torque sign/direction while in drive, low, and reverse.

Complete experimental results for 415 V, two-pole, 22-kW induction machine are presented. The machine needs to be operated in an unpowered, coasting mode as mentioned previously. Coast-down (a.k.a. run-down or spin-down) is defined to be the behavior of the rotating system that goes unpowered until the system comes to rest. When the power to the rotating system is cut off, the energy dissipation in the system due to the frictional effects of bearings, windage, *etc.* causes deceleration of the rotor until it comes to rest. Therefore, the entire behavior of the system, from the instant of power cut off to the instant of precise halting, is called coast-down phenomenon, and the duration of this phenomenon is called the coast-down time [25].

**Figure 10.**
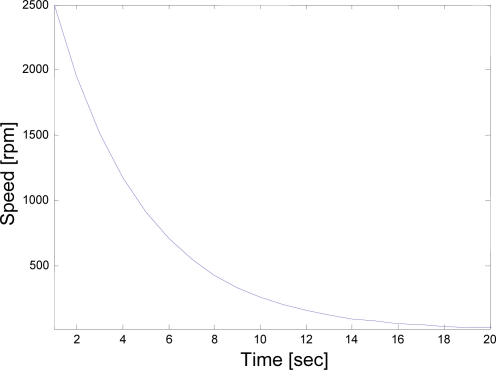
Experimental coast-down curve.

An experiment was carried out in which the motor was firstly brought up to the vicinity of synchronous speed of 3000 rpm and then brought down or launched from a measured speed of 2500 rpm at 50-Hz supply (a.k.a. cut-off speed) to 0 rpm in 20 seconds as can be seen in Figure 10.

Figure 10 illustrates the variation of speed with time for the rotating system obtained during the coast-down test of a 415 kV, two-pole, 22-kW induction machine. As a matter of fact, this is the figure of the rpm vs. time for the filtered data after several undesired signals such as the amount of random noise and external disturbance signals have been removed from the raw speed data of the coast-down through the use of adaptive average filtering algorithms to preserve the accuracy of the data over the full speed range between the synchronous speed and zero during the coast-down. Raw speed data was collected at a sampling rate of 4000 samples per second (*i.e.*, the frequency was 4 kHz).

In the following part, the induction motor of the actual system was used, and the algorithm for the wheel profile irregularities was implemented to obtain the compensation factors that were based on the chi-square test. These factors were iteratively determined during in-use machine operation and were updated on-line.

**Figure 11.**
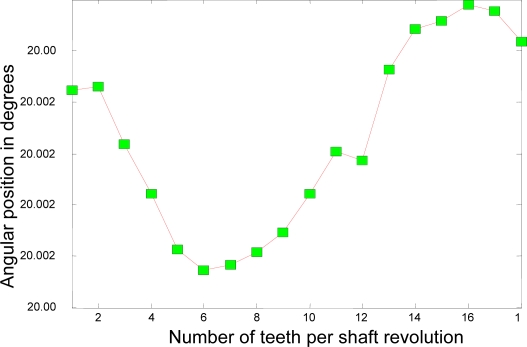
Angular delta shaft position vector corresponding with unique set of compensation factors.

**Figure 12.**
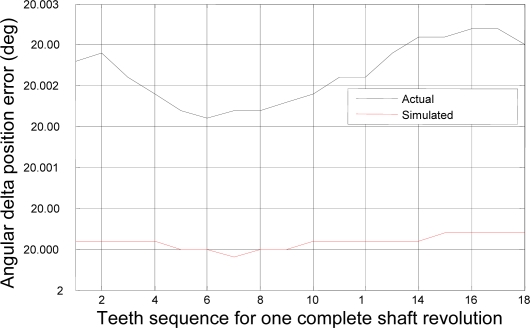
Angular delta shaft position error vector for both simulated and actual systems after compensation.

Now, the experimental results for the actual system of the 22-kW induction motor as based on the coast-down data above are presented. Figure 11 depicts the rotational angular delta position vector after the compensation algorithm is applied. Figure 12 shows the angular delta shaft position error vector for both the simulated and actual systems after the compensation algorithm is applied.

The most noticeable distinction between Figures 4 and 11 is the accuracy of the angular position. The range of the angular position in Figure 4 varies from 20.0004 degrees to 20.0008 degrees; however, the range of the position in Figure 11 varies from 20.002 degrees to 20.004 degrees. This distinction suggests that the range of the position for the simulated system has an accuracy of, at least, 4 digits whereas the range of the position for the actual system, at least, 3 digits. The ability of the measurement to match the actual value of the position tells us that the simulated system is at least one digit more accurate after the decimal point than the actual system. However, two digits of trailing zeros after the decimal point for the actual system are still sufficient for the effective compensation. This difference is mainly due to the frictional effects of bearings, windage, *etc.* as expected.

It is observed from the experiment that the proposed algorithm compensates for the toothed wheel profile irregularities thus position errors accurately and not only allows for changing velocity and not only handles geometric constraints, but also is capable of compensating for the irregularities quickly and efficiently. This is very important performance criterion in the position error compensation problem. The method also presents many advantages of which some are the reduced risk of the control system’s failure or malfunction, and the computationally simple implementation without extensive memory or processing requirements. The compensation factors are obtained iteratively without the need for a look-up table or stored compensation factors from previous operations or experience.

## 6. Conclusions

A VR sensor has been used to correct the position of the electric machine of a parallel HEV system, mainly because it may still become critical in the operation of HEVs to avoid possible vehicle failures during the start-up and on-the-road, especially when the electric machine is used with an internal combustion engine. Both simulated and actual results have been presented to demonstrate the accuracy of the method. Specifically, the actual results show the effectiveness of the presented method and that the method is capable of compensating for the irregularities and the compensation was rather accurate and the algorithm was rather quick and efficient. In the future, the plan is to extend this study to investigate the performance of the proposed compensation method under the transient operating conditions, which is usually considered for the HEVs that could only use the motor to assist or start the engine.
